# Thrombotic thrombocytopenic purpura patients' attitudes toward depression management: A qualitative study

**DOI:** 10.1002/hsr2.136

**Published:** 2019-08-29

**Authors:** Deirdra R. Terrell, Eleni L. Tolma, Lauren M. Stewart, Erin A. Shirley

**Affiliations:** ^1^ Department of Biostatistics and Epidemiology, Hudson College of Public Health University of Oklahoma Health Sciences Center Oklahoma City OK; ^2^ Department of Medicine, College of Medicine University of Oklahoma Health Sciences Center Oklahoma City Oklahoma; ^3^ Department of Social Behavioral Sciences, Faculty of Public Health Kuwait University Health Sciences Center Jabriya Kuwait

**Keywords:** ADAMTS13, depression, qualitative research, thrombotic thrombocytopenic purpura, TTP

## Abstract

**Background and aims:**

Thrombotic thrombocytopenic purpura (TTP) is a rare disorder characterized by acute episodes of systemic microvascular thrombosis; TTP is more common in adults, women, and African‐Americans (Blacks). Our Oklahoma TTP Registry documented that survivors have an increased prevalence of depression compared with the general population; however, many patients' depression remains untreated. Moreover, studies identifying attitudes toward depression management are lacking. The objective of this study was to identify TTP patients' attitudes towards pharmacotherapy. As a secondary question, we explored attitudes towards counseling.

**Methods:**

We interviewed TTP patients with major depression who had experience with different management strategies (previous/current pharmacotherapy treatment versus no pharmacotherapy treatment). Eligibility criteria included (a) age > 18 years, (b) ADAMTS13‐deficient TTP, (c) enrolled in the Oklahoma Registry, and (d) moderate/major depression on either the Beck Depression Inventory II or Patient Health Questionnaire from 2004 to 2012. Qualitative purposive sampling was used to interview patients with a range of experiences with TTP and depression symptom management. Our study was based on the theoretical framework of the Theory of Reasoned Action. Patients were asked about their views on depression (attitudes), their family and friends' views (social norms), and ways they cope with depression.

**Results:**

Semi‐structured interviews were conducted between June and October 2013. Data saturation was achieved after interviewing 16 patients (nine, pharmacotherapy and seven, no pharmacotherapy). The majority (88%) were women; 56% were Black, and the median age was 49 years. Patients in both groups believed TTP was life altering and traumatic and that counseling improved depressive symptoms. However, the pharmacologic group believed medication improved one's quality of life, whereas the no pharmacotherapy group was not sure pharmacotherapy was effective and expressed fears related to potential addiction and side effects. When asked about cultural views to depression management, many Black patients stated that in the Black community, a person is taught to deal with his/her emotional issues instead of asking strangers for help.

**Conclusion:**

Ensuring effective depression management is a critical part of TTP care. Understanding attitudes toward management will assist in tailoring patient discussions.

## 1 INTRODUCTION

Acquired autoimmune thrombotic thrombocytopenic purpura (TTP) is a rare disorder characterized by acute episodes of systemic microvascular thrombosis. Without prompt recognition and plasma exchange treatment (standard therapy), TTP has a mortality of 90%,^1^ but with early recognition and treatment, 78% to 90% of patients will survive beyond their acute episode.^2^, ^3^ Modern understanding is that TTP is caused by autoantibody‐induced severe deficiency of a plasma enzyme, *a* *d*isintegrin *a*nd *m*etalloprotease with *t*hrombo*s*pondin 1‐like domains (ADAMTS13)^2^, ^4^, ^5^, ^6^. TTP is diagnosed based on clinical suspicion and the presence of microangiopathic hemolytic anemia and thrombocytopenia without an alternative diagnosis.^3^, ^5^ The age‐sex‐race adjusted annual incidence estimates are 2.9 per 1 000 000 adults, 8.8 per 1 000 000 Blacks or African‐Americans (referred to as Black for the remainder of this paper), and 1.2 per 1 000 000 non‐Blacks.^7^ TTP is more common in women, occurs during the child‐bearing years, and rarely occurs in children less than 18 years of age.^3^, ^7^

It has been documented that patients who have recovered from acquired TTP have multiple health problems that occur following recovery, including major depression.^8^, ^9^ Major or moderate depression has been reported in approximately 40% of TTP survivors.^8^, ^10^ However, despite an increased prevalence of depression in TTP survivors when compared with the general population,^9^, ^10^, ^11^ TTP patients' depression often remains untreated.^11^ This is significant, because major depression is projected to be the second leading cause of disability worldwide by the year 2020.^12^ Untreated depression can be chronic, and many individuals will experience recurring episodes, leading to substantial impairments in their ability to attend to everyday responsibilities.^13^ Additionally, major depression impacts a person's health‐related quality of life, and it is a risk factor for suicide.^14^, ^15^, ^16^ Management options for treating major depressive disorder include pharmacotherapy, psychotherapy (including counseling), or a combination of both, taking into account patient's preferences.^16^

Although pharmacologic treatment and counseling can be effective in reducing depressive symptoms, the mental health needs of individuals within the United States are underserved.^17^, ^18^ Patients with depression sometimes struggle to identify with a medical depression diagnosis, and attitudes toward depression treatment often differ.^16^ Studies evaluating attitudes to receiving management for major depression in TTP patients are lacking. Recognizing and understanding TTP patients' attitudes towards depression management may help a larger number of patients access proven therapies to relieve depression symptoms.^19^

Therefore, our primary objective for this study was to identify TTP patients' attitudes regarding pharmacologic treatment for depression. As a secondary question, we explored themes related to TTP patients' attitudes regarding counseling as a treatment for depression. To achieve the stated objective, we interviewed TTP patients with major depression who had experience with different depression management strategies. The theoretical framework of our study was guided by the Theory of Reasoned Action, which is a value expectancy model of decision making that states that a person's behavior is determined by intention and a person's intention is determined by (a) a person's attitude toward the behavior and (b) how others close to them view the behavior (subjective norms).^20^ The Theory of Reasoned Action has been successfully used as a framework in studies of depression management to identify potentially modifiable factors such as patient attitudes (related to the effectiveness of depression treatment or attitude toward a type of depression treatment) and social norms (a loved one's views toward depression treatment impacted patient adherence to treatment) on depression management. This is important because these factors (attitude or social norms) have the potential to be modified by education or experience.^21^, ^22^, ^23^

## 2 METHODS

### 2.1 Study design

A qualitative descriptive study was conducted in order to understand TTP patients' attitudes toward depression management. The methodologic design of our study was guided by the Theory of Reasoned Action,^20^ because it has been successfully used as a framework in studies of depression management.^21^, ^22^, ^23^ Although sample size cannot be predetermined in a qualitative study, according to the theory, the goal is to conduct interviews with approximately 12 to 20 individuals,^24^, ^25^ about half of whom have performed the behavior under investigation (ie, use of pharmacologic treatment) and half of whom have not performed the behavior.^25^ Interviews were conducted until no new comments were heard related to our key questions of interest, which is defined as data saturation.^24^, ^26^

The first author consulted with both a TTP expert and a qualitative expert to develop two similar interview guides (one for patients with current/previous use of pharmacologic treatment for depression and one for patients who had never used pharmacologic treatment). The interview guides were guided by the Theory of Reasoned Action.^20^ The interview guides were then pilot‐tested with three TTP patients and revised accordingly. The pilot test interviews were included in the analysis.

Patients were asked about their views on depression, their family and friends' views, and ways they cope with depression (Table 1). Specifically, patients were asked if there were any benefits or disadvantages associated with treating depression with medication or counseling (attitudes). Participants were also asked how family or friends feel about people taking medication or going to counseling for depression (subjective norms). Subjective norms were extended to include questions on cultural views of depression because previous studies have shown Blacks and Hispanics have more negative views regarding acceptance of pharmacologic treatment for depression management compared with Whites.^23^

**Table 1:** Semi‐structured interview guide

• What are the first 5 to 10 things that immediately come to your mind when I say the word “depression”?
• Can you describe to me a time in your life when you felt depressed?
• Who are some of the people that you trust that you talk to about your depression?
• How do you think your depression is viewed by your family?
• How do you think your depression is viewed by your friends?
• There are a lot of ways to deal or cope with depression. What are some of the ways you personally deal or cope with your depression?
• One of the ways people treat depression is by talking with a professional. What do you think about treating depression with counseling?
• (Attitude toward counseling) What, if any, are the some of the benefits of talking with a counselor for depression?
• (Attitude toward counseling) For you, what, if any, are the negatives of talking with a counselor for depression?
• (Subjective norms toward counseling) How do your family/friends feel about people going to a professional counselor for depression?
• We have in our records that you have/have never taken medicine for depression. Is that true? Can you tell me some reasons? Have you ever started and stopped? Tell me more about that.
• (Attitude toward pharmacologic treatment) What, if any, are the benefits of treating depression with medicine?
• (Attitude toward pharmacologic treatment) Now, what, if any, are the negatives of treating depression with medicine?
• (Subjective norms toward pharmacologic treatment) How do your family/friends feel about people taking medication for depression?
• Can you describe for me certain things or circumstances where you would get some treatment for your depression?
• (Subjective norms toward depression) Can you tell me how you think people who are around the same age as you or who grew up around the same time as you think about a person who has depression?
• (Subjective norms toward depression) How do you think people who are the same race or culture as you think about a person with depression?
• (Subjective norms toward depression) In what ways do you think thoughts or opinions about mental health or depression as you were growing up influences the way you think about it?
• How do you think those views impact or affect someone with depression trying to get help?
• Is there anything else you would like to tell me about depression or treating depression?

*Note.* Attitude is defined as the positive or negative evaluation of performing a behavior. Subjective norms are defined as the social influences and normative pressures an individual may perceive.

### 2.2 Oklahoma TTP‐HUS Registry

The Oklahoma TTP‐Hemolytic Uremic Syndrome (HUS) Registry is a prospective, population‐based inception cohort of all consecutive patients identified by a request to the Oklahoma Blood Institute (OBI) for plasma exchange treatment for a diagnosis of TTP or HUS. The OBI is the sole provider of plasma exchange treatment for all hospitals in 58 of Oklahoma's 77 counties. Plasma exchange treatment is the standard care for all patients diagnosed with TTP^1^, ^27^; therefore, all patients with a suspected initial diagnosis of TTP are enrolled in the Registry.^28^ Since November 1995, serum samples have been collected immediately before the first plasma exchange, and ADAMTS13 activity levels are tested by two methods.

ADAMTS13 (also known as von Willebrand factor [VWF] cleaving protease) activity was measured by both quantitative immunoblotting of degraded, plasma‐derived VWF substrate and fluorogenic assay using FRETS‐VWF73 substrate, Pefabloc SC (Sigma Aldrich‐catalog #76307). Patients are designated as having severe ADAMTS13 deficiency if ADAMTS13 activity is less than 10% of the ADAMTS13 activity in normal pooled human plasma.^2^ Patients were diagnosed as having an acquired etiology of TTP if their ADAMTS13 activity was less than 10% activity at the time of their initial episode or at the time of a relapse.^29^

### 2.3 Depression

Beginning in 2004, all patients who were in clinical remission from TTP have been asked to participate in annual in‐person evaluations. The Beck Depression Inventory (BDI)‐II was administered during the years 2004, 2006, and 2009 to 2011. The BDI‐II is a validated, self‐administered depression screening instrument that has 21 statements related to depressive symptoms from the previous 2 weeks, and the scoring options range from 0 to 3. Patient scores from 0 to 13 are interpreted as no or minimal depression, 14 to 19 as mild depression, 20 to 28 as moderate depression, and scores > 28 are interpreted as severe depression.^30^

The Patient Health Questionnaire (PHQ)‐9 was administered in 2012 and is a validated, self‐administered depression screening instrument that has nine questions evaluating depressive symptoms over the previous 2 weeks.^31^ Major depression is diagnosed when greater than or equal to 5 symptoms are present for at least “more than half the days” and one of those symptoms has to be either depressed mood (“feeling down, depressed, or hopeless”) or anhedonia (“little interest or pleasure in doing things”). Other depression is diagnosed if 2 to 4 depressive symptoms are present for at least “more than half the days” and one of those symptoms has to be either depressed mood or anhedonia. If the final question, “thoughts that you would be better off dead or of hurting yourself in some way,” is present during the previous 2 weeks, then it is also counted as one of the symptoms.^31^

A study coordinator scored the depression instruments immediately during the patient's annual evaluation, and results were given to the patient. All patients with moderate or major depression were given depression referral information. Additionally, we notified their hematologist and/or primary care physician with their depression results.

For this study, patients with severe depression with the BDI‐II screening test are described as having major depression, to maintain consistent terminology between the BDI‐II and the PHQ‐9.

### 2.4 Participants

Eligibility criteria included (a) age > 18 years of age, (b) survived previous episode of acquired ADAMTS13 deficient TTP, (c) enrolled in the Oklahoma Registry, and (d) received at least one depression screening result scored as moderate or major depression on either the BDI‐II or PHQ‐9 from 2004 to 2012.

TTP patients with moderate or major depression were then placed into two groups: (a) patients who never had pharmacologic treatment for their depression and (b) patients with previous/current pharmacologic treatment for their depression. Previous/current pharmacologic treatment was based on a medical chart review. Patients in the no pharmacologic treatment group included patients who had directly refused pharmacologic treatment for depression (these patients attended a consultation with their doctor and were prescribed pharmacologic treatment to which they either directly refused or decided not to take it), and it also included patients who never followed up with our referral to consult with a physician based on their depression screening score. As all patients (and their physicians) had been informed of each depression screening result from our annual evaluations, patients who never had pharmacologic treatment for their depression represented patients with untreated depression, and patients with previous/current pharmacologic treatment for their depression represented patients with treated depression.

Patient attitudes regarding pharmacologic treatment for depression were compared between these two groups. Previous or current counseling history was not available in the Registry medical records because it is not systematically assessed. Therefore, as a secondary question, we explored themes related to TTP patients' attitudes regarding counseling as a treatment for depression.

A nonprobabilistic purposive sampling approach was utilized to select individuals from whom the most could be learned.^32^ To ensure diversity and a range of experiences with TTP and depression symptom management, the goal was to include at least one male and one Black participant within both groups. A study using data from the United States National Ambulatory Medical Care Survey showed that racial disparities (Blacks and Hispanics compared with Whites) exist in both the diagnosis of depression and the utilization of pharmacologic treatment.^33^ In addition, a study reported that Blacks and Hispanics are less likely to perceive a need for mental health treatment as compared with Whites even after adjusting for severity of mental illness.^34^

Fifty‐six patients in clinical remission from acquired TTP had been screened for depression between 2004 and 2012; 50 patients were alive in 2013 when this study was performed; 24 of these patients had a score of moderate or major depression on at least one assessment. Our goal was to interview patients until we reached data saturation, defined as redundancy in the comments.^24^, ^26^ Given that the principal investigator knew the majority of these patients from previous Registry studies, we used a simple random sample program (SAS version 9.3, SAS Institute, Cary, NC) to determine the order in which the eligible patients should be approached and asked about their willingness to participate in the approximately 60‐ to 90‐minute interview. Of the patients who were randomly selected to be approached, none of them refused our invitation for an interview. However, two patients (one male in the no pharmacologic treatment group; one female in pharmacologic treatment group) originally selected by the random number program were unable to be contacted due to inaccurate contact information and were thus replaced with other patients selected by the random number program.

### 2.5 Data collection

The history of TTP was obtained from medical chart review, patient demographics were obtained from self‐reported data collected on the day of the interview, and self‐reported information on mental health disorders was obtained from previous annual evaluations where the information was systematically collected. Semi‐structured interviews were used because they allow for flexibility in exploring participant experiences and attitudes related to depression management.^32^

Interviews were conducted from June to October 2013, were 60 to 90 minutes in duration, and were digitally recorded. Rigor was enhanced by having DRT conduct all the interviews, and the majority of the patients knew her from their participation in previous Registry research studies; DRT is a doctoral level epidemiologist who has been a member of the TTP Registry research team since 2001. She has been trained in qualitative methods and has experienced conducting qualitative interviews.

The interviews were conducted in a private office at the University of Oklahoma Health Sciences Center in Oklahoma City, Oklahoma, on the same day as the patient's annual, in‐person evaluation. To enhance accuracy of the transcripts, a notetaker was present in the room for all interviews to record key responses and body language. Family members and friends, if present, were asked to leave the room prior to the start of the interview. In addition, participants were informed of the goal of the study in detail prior to the start of the interview. Interviews were conducted until we began to hear redundancy in the comments related to our key questions of interest, which is defined as data saturation.^24^, ^26^ Repeat interviews were not conducted. Transcripts were not returned to the participants for comment/correction and participants were not asked to provide feedback on the results.

### 2.6 Analysis

Data were analyzed according to the framework of the Theory of Reasoned Action. We also looked at themes and key ideas outside of the framework of the Theory of Reasoned Action. Each interview was transcribed verbatim. The accuracy of the transcriptions was verified by DRT prior to content analysis by comparing the transcribed content against the recorded interviews. Content analysis was conducted by four researchers trained in qualitative research methodology (DRT, LMS, EAS, and ELT) and facilitated by using the QSR NVivo 10.0 software. Initially, the research team independently reviewed two transcripts and assigned codes (categories) on specific concepts of interest. Codes were finalized with the agreement of all the team members and summarized into a codebook that further guided the analysis. The codebook contained 17 codes (or nodes) and their perspective definitions. One of the researchers (EAS) then coded each transcript by using QSR NVivo 10.0 software. To ensure that the coding was done consistently, the first two coded transcripts were reviewed by the lead investigator (DRT). Once coding was completed, with the use of the computer software, we retrieved the participants' comments that were directly related to purpose of the study and the Theory of Reasoned Action. During the analysis, we identified similar patterns of beliefs across all interviews, unique insights, and the underlying meaning of the participants' comments. Once the analysis was completed individually by each of the four researchers, we met and discussed the findings. By discussing the results as a group, we attempted to remove personal bias that might influence the interpretation of the results. Discrepancies were discussed and resolved as a team, and after complete consensus, we identified broad themes and key ideas.

Themes were defined as clusters of codes representing an idea mentioned by at least half of the participants within each group (pharmacologic treatment vs no pharmacologic treatment). Key ideas were novel insights that were deemed important findings to include by the research team even though they were not mentioned by at least half of the participants.^35^ We used the Consolidated Criteria for Reporting Qualitative Research (COREQ) checklist to promote a comprehensive report of the methods and findings.^36^

### 2.7 Compensation

All participants were compensated $40.00 for participation.

### 2.8 Ethical approval

Patients gave informed consent in accordance with the Declaration of Helsinki and were informed and consented to the interviews being digitally recorded. This study was approved by the ethical approval granting board at the University of Oklahoma Health Sciences Center, Oklahoma City, Oklahoma.

## 3 RESULTS

### 3.1 Patient demographics

**Figure 1:**
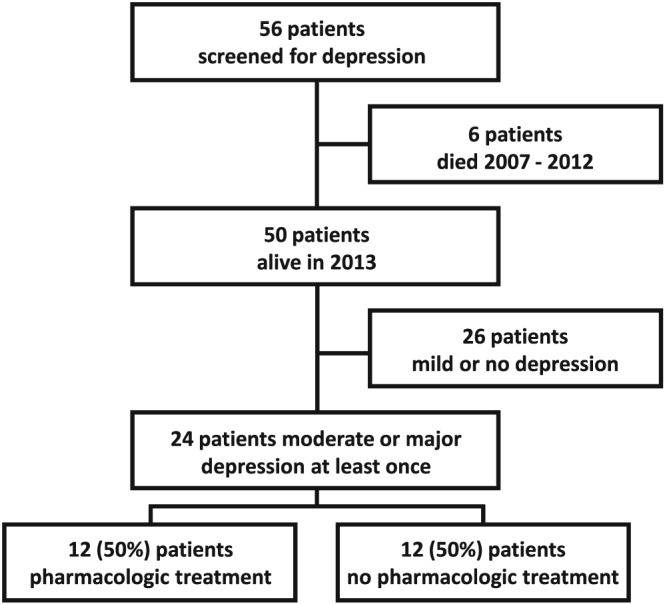
Acquired autoimmune thrombotic thrombocytopenic purpura (TTP) patients eligible for patient interviews

Fifty‐six patients in clinical remission from acquired TTP had been screened for depression between 2004 and 2012; 50 patients were alive in 2013 when this study was performed; 24 of these patients had a score of moderate or major depression on at least one assessment. Of the 24 patients, 12 (50%) had taken pharmacologic treatment for their depression and represented patients with treated depression, while 12 (50%) patients had never taken pharmacologic treatment and represented patients with untreated depression (Figure 1).

Data saturation was achieved after interviewing 16 patients. The goal of the qualitative purposive sampling was to interview patients with a diversity of experience. The demographics of the interviewed patients were similar to what we would expect for this disease^7^: 14 patients (88%) were women, nine (56%) were Black, and the median age was 49 years (range: 27‐69) (Table 2). Demographics of the patients who were not interviewed were similar to the interviewed patients except the eight patients who were not interviewed were 75% White. The interviewed patients were a median of 7 years from their most recent TTP episode (range: 1‐16 years), five (31%) had experienced at least one TTP relapse, and six (38%) had also received rituximab treatment for their disease (Table 2).

**Table 2:** Characteristics of patients participating in semi‐structured interviews

Demographics (N = 16)	
Self‐reported race (n, % Black)	9 (56)
Sex (n, % women)	14 (88)
Age in years (median, range)	48.5 (27‐69)

Education (N = 16)	
High school graduate (n, %)	7 (44)
More than high school graduate (n, %)	9 (56)

Marital status (N = 16)	
Never married (n, %)	4 (25)
Married (n, %)	7 (44)
Divorced (n, %)	3 (19)
Widowed (n, %)	2 (12)

Thrombotic thrombocytopenic purpura (TTP) history (N = 16)	
TTP relapse (n, % yes)	5 (31)
Rituximab treatment (n, % yes)	6 (38)
Years since last TTP episode median years (range)	7 (1‐16 y)

Mental health (N = 16)	
Self‐reported history of mental health diagnosis prior to TTP (n, % yes)	5 (31)
Self‐reported family history of mental health diagnosis (n, % yes)	7 (44)

Nine of the 16 patients had a history of taking pharmacologic treatment to manage their depression, and four of these patients were taking pharmacologic treatment on the day of the interview. Seven of the 16 patients did not have a history of using pharmacologic treatment to manage depression according to our records; however, during the interview, one patient described that they had recently been prescribed sertraline and hydroxyzine for anxiety. The patient stated they had taken the medication for about 2 weeks but stopped taking it and was currently not on any medication (for analysis, this patient remained in the no pharmacologic treatment group). We did not systematically collect information on current use of counseling. Five patients had a self‐reported history of a mental health diagnosis (three, depression; one, anxiety; and one, borderline personality disorder) prior to their first TTP episode (Table 2). On the day of the interview, eight patients had PHQ screening evaluations indicating major depression (two, no pharmacologic treatment group and six, pharmacologic treatment group), one patient had a score indicating other depression (no pharmacologic treatment group), and seven patients had scores indicating no depression (three, pharmacologic treatment group and four, no pharmacologic treatment group).

### 3.2 Qualitative results: Themes and key ideas

Patients' beliefs regarding pharmacologic treatment and counseling were compared between patients who had taken pharmacologic treatment for their depression and patients who had never taken pharmacologic treatment for their depression. Qualitative results are presented according to the framework of the Theory of Reasoned Action and focused on themes and key ideas related to the patients' attitude toward the use of pharmacologic treatment and counseling and their beliefs on how others close to them viewed depression management (subjective norms). Illustrative quotes from each group are also presented.

### 3.3 Attitudes: Pharmacologic treatment

The majority of patients who had never tried pharmacologic treatment were unsure if a medication for depression could actually work to relieve their depressive symptoms. One patient also expressed concern about what would happen when the medication “wore off,” wondering whether their depressive symptoms would then come back even stronger. Conversely, the majority of the patients in the pharmacologic group viewed medication positively and believed medication was effective in treating their depressive symptoms and improving their quality of life. The majority of these patients described greater emotional stability, the ability to connect with people, make better decisions, and the ability to socialize again when taking pharmacologic treatment, as illustrated by this quote:

Got me on meds and my life has been a lot better emotionally since then. At least I've been able to face emotional challenges better than perhaps I did. (White female, 52 years old, pharmacologic treatment)

When asked about the negatives of treating depression with medication, many patients in the pharmacologic group experienced a side effect known as emotional numbing, which they described as feelings of not caring about anything and the inability to get excited about things. These patients went on to describe the impact of emotional numbing on their relationships and the associated difficulties with taking care of a home and/or small children. Some patients expressed this particular side effect of emotional numbing was sometimes severe enough to make them stop taking their medication completely or to request to be put on a different medication. Illustrative quotes are as follows:

I felt like a zombie on Cymbalta … I'd almost rather be sad than not have any feelings at all (White female, 54 years old, pharmacologic treatment)

… could sit on the couch and not care if the house burnt down when I was sitting in it … and I have two kids that I have to take care of (White female, 33 years old, pharmacologic treatment)

Although patients in the no pharmacologic treatment group did not have any actual experience of taking antidepressant medication, they expressed fears of becoming addicted to a medication and also fears of potential side effects that would alter how they relate to their world and their families. A few patients described how they have seen family members or friends who were on antidepressants no longer act like themselves. These fears were enough to keep them from trying a medication for depression. An illustrative quote is as follows:

I don't like mind altering stuff … being out of my normal state, I don't want, you know, yeah, I like to think clearly (White male, 42 years old, no pharmacologic treatment)

### 3.4 Attitudes: Counseling

Overall, patients in both groups believed counseling was a good tool to use in depression management. Although we did not directly ask patients if they had ever been to counseling, some patients described personal experiences of how counseling had improved their lives. For example, many patients stated that counseling allowed one to vocalize the problems and there was “… a benefit of getting it out and letting it out and speaking it.” Patients identified that the process of vocalizing the problem allowed for introspective thought. Additionally, a few patients in both groups stated that one benefit of counseling was being able to discuss problems with someone who could provide an objective alternative perspective. Illustrative quotes are as follows:

… Ooh, you get your life back. Yeah, you can be happy, you can smile again, I think. You know, now I'm not out there like I was, but I'm getting there. I can see myself coming out and seeing the sunshine, or opening my blinds. Mhm. I can sit on the porch. Yeah, I see changes. (56 year old, Black female, pharmacologic treatment)

Uhm, just to keep you from having a breakdown outside, like it's like the room is the place that you can break down and the world doesn't have to see it, but when you come out of the room, it's like, okay, I'm done with crying about that, so, you can actually discuss it without breaking down every time. (32 year old, Black female, no pharmacologic treatment)

Themes from both groups related to barriers for utilizing counseling as a depression management option included a mistrust of the health care provider's ability to improve their depressive symptoms. Again, although not directly asked, it seemed from their statements that mostly patients who had never had counseling feared that counselors might just “tell you what you want to hear” or that they did not genuinely care about you but only cared about financial reimbursements. One patient described it this way:

… and ah, the negatives about it is that some of them are overpriced you know, it's all about the money, you know, it's all about the money, you know, maybe you cared at one time about doing somebody well but maybe not no more. Maybe I'm wasting my money and my time, are you really helping me? (42 year old, White male, no pharmacologic treatment)

A key idea in both groups was that a history of a negative experience with counseling made it more difficult for a person to want to try it again. These patients described personal experiences and stated sometimes they felt like they just could not connect with the counselor on a personal level and that a personal connection was essential for counseling to be effective.

### 3.5 Subjective norms (family/friends' views): Pharmacologic treatment

We wanted to understand the social influences and normative pressures patients experienced regarding depression management. A key idea in the pharmacologic group was that their family and friends were supportive. Patients stated that their family understood and viewed depression like any other illness (ie, diabetes or hypertension) and viewed depression medication as something that they needed to take in order to be better and they supported and encouraged them to take it:

… she's good with me being on medication, she'd much rather me be up out of my bed and wanting to do stuff, wanting to be happy because you know the opposite is not very much fun at all. (57 year old, White female, pharmacologic treatment)

However, a key idea in the no pharmacologic treatment group was that friends/family were not supportive of their loved one taking a medication for depression. Patients stated when they had been involved in conversations regarding either the possibility of themselves taking a medication for depression or talking about someone else taking a depression medication, and the views were that medication was unnecessary. In fact, their family and/or friends voiced concern regarding medication dependency and expressed there were alternative ways to handle depression. An illustrative quote is as follows:

Oh, they're against it … they don't like seeing everybody, you know, have to be dependent on medication to get well or something like that, you know? (67 year old, Black female, no pharmacologic treatment)

### 3.6 Subjective norms (family/friends' views): Counseling

A key idea in the no pharmacotherapy group was that their family or friends would support them going to counseling for depression. They stated they believed those close to them would be happy that they are getting help and getting better. However, in the pharmacotherapy group, half the patients believed their friends or family would support them going to counseling, but half of the patients kept it from their friends or family or thought they would be against the idea. The patients who did not tell their family or friends were afraid their loved ones would view them as “crazy” or view getting help as a sign of weakness.

We also extended social norms to include cultural views of depression. A theme identified in Blacks and Whites was the belief that there was a stigma associated with having a diagnosis of a mental health disorder. Some patients did not tell others they felt depressed because they were worried that people would think they were weak or treat them differently. Furthermore, some patients who had shared with others they suffered from depression said they felt like people thought they were crazy.

And you know I just, you know my my my boss is one of my people on Facebook and I finally had to break down and tell him what's goin on and he kinda makes jokes like me goin to the looney bin and stuff (28 years old, White female)

My mom thinks I'm crazy, you know, she's like ‘that's some bullshit’ you know? That's some bullshit, that's some of that White folk stuff up there you know?...what do you got to complain about, you know? We used to not be able to drink out of the same water fountain, you know, you just need to concentrate on your blessings and move forward, you ain't got nothing to be depressed about (41 years old, Black female)

Many Black patients said they often tried to hide their depression from their friends and loved ones; however, often, their close family recognized their depression and tried to cheer them up. Many Black patients went on to state that in the Black community, a person is taught to deal with his/her emotional issues instead of asking strangers for help. An illustrative quote is as follows:

In the Black community, you're taught that whatever issues you just deal with them. You make the best of any situation, so to me it's harder to talk about it in our community (32 years old, Black female)

### 3.7 Additional barriers to depression management

When asked what circumstances would need to change in order for the patient to get treatment for their depression, a key idea was the belief that their depression was not severe enough to get help. Patients stated their depression was not “that bad yet.” When asked to describe how severe their depression would need to be before they would consider counseling or pharmacotherapy, patients stated they would have to “wake up depressed” or it would have to be a daily occurrence. Patients also stated another measure of severity was if the depression felt like they could no longer handle it themselves (utilizing their own coping mechanisms such as talking with family or friends). An additional barrier expressed by a few patients was that counseling and/or pharmacotherapy was expensive regardless of insurance status.

### 3.8 Impact of TTP on the patients' lives and the role of the hematologist

Important insights about surviving TTP were also identified. Specifically, a theme that arose was that surviving TTP was life altering and traumatic. Although some patients expressed that during their acute episode they feared they would die, it was actually the residual symptoms after their TTP was in remission (such as fatigue and mild cognitive impairment) that changed their lives. Many patients expressed the belief that surviving TTP had changed them both mentally and physically. Furthermore, patients described that fatigue made it difficult for them to do activities with their loved ones or hobbies they used to enjoy and, consequently, resulted in social isolation.

Many Black patients believed that dealing with the residual symptoms of TTP (fear of relapsing, fatigue, and mild cognitive impairment) was related to their depression. These patients did not understand what triggered the TTP the first time, and they did not want to get it again, so patients would isolate themselves for fear of others getting them sick. Additionally, a few patients stated they often felt “worthless” because they could no longer do the things (ie, taking care of their children or doing home improvement projects with their spouse) the same way they were doing them before they got sick, and needing additional help to perform their daily activities made them sad. Illustrative quotes were as follows:

This was extremely, extremely uh life altering. It was a huge deal … I'd never cognitively gotten back to where I was … I was one of those people that went and ran seven days a week. I stayed on the go, all the sudden everything, I just could not, and I still can't do it, and it took me a long time to accept that. (41 year old, Black female, pharmacologic treatment)

My body wasn't holding this on and if I do this and that and that and this, I'mma die. It freaked me out a lot, so I went into depression. And you know, like, panic attacks when you go to stores … couldn't let anyone come to visit me because I was afraid to trigger that TTP … I felt like if I go to sleep, I wasn't going to wake up.” (56 year old, Black female)

Another key idea discovered during the interviews was the vital role the primary hematologist plays in these patients' lives. Given that TTP is a rare condition that can be fatal, patients often credit their hematologist with saving their lives. Therefore, hematologists' recommendations are especially critical during both the acute episode and during remission. In fact, the discussion of depression with a hematologist was instrumental in the initial treatment of one patient's depression:

My hematologist said, ‘Hey, how are you doing?’ Off the cuff, and I just mentioned, ‘well, I just, uhm, I'm okay, I'm okay. I've just been a little depressed.’ And instead of him blowing it off … he grabbed onto that and thankfully made an appointment for me. My hematologist said, ‘I know this guy (psychiatrist), he understands ‘bout this stuff.’ I believed the hematologist because he had saved my life … I honestly went to see this guy [psychiatrist] the one time as a favor to the hematologist. (52 year old, White female, pharmacologic treatment)

## 4 DISCUSSION

In the Oklahoma TTP Registry, we have previously documented that the point prevalence of severe depression in TTP patients was significantly greater than the point prevalence of severe depression in the US population after adjusting for age, race, sex, and body mass index.^9^ Chaturvedi et al confirmed an increased prevalence in their TTP patients and also reported that depression is suboptimally treated in these patients.^11^ In the current study, we explored TTP patient attitudes, friend/family views, and cultural norms regarding seeking depression management. The current study is significant because it is the first study to ask about the experience of depression from the TTP patient perspective in a qualitative manner.

Survivors of TTP with a history of pharmacologically‐treated depression believed medication was effective as a management option. However, our TTP patients acknowledged that a major barrier to pharmacologic treatment is a side effect known as emotional blunting. Patients who had not tried pharmacologic treatment for their depression expressed barriers related to a fear of potential side effects and addiction/dependency on a medicine. It is important to acknowledge these fears during the discussions with patients when considering prescribing pharmacotherapy for depression. In our study, we also explored if there were any cultural barriers related to depression. Black TTP patients were more likely than White patients to state a cultural barrier to depression treatment. Many Black patients stated that in the Black community, a person is taught to deal with problems on their own and not seek help outside of one's family; therefore, it was harder to talk about depression with strangers. In terms of counseling, regardless of pharmacologic treatment history, TTP patients expressed counseling was an effective depression management option. However, they believed that for counseling to be effective, it was essential to have an interpersonal connection with the mental health professional, as well as establishing confidentiality between the two parties.

The views from our TTP patients are similar to what has been reported by others who have studied persons with pharmacologic‐treated depression. Similar to our study, a previous study found patients who had taken antidepressants in the past had more favorable attitudes toward pharmacologic treatment.^19^ Furthermore, the side effect from pharmacologic treatment of emotional blunting reported by the TTP patients is a commonly reported side effect of selective serotonin reuptake inhibitors.^37^ Additionally, fears related to pharmacologic treatment are similar to previous studies done with participants with conditions other than TTP. A focus group of Black women, without TTP, with major depressive disorder, described fears of pharmacologic treatment related to unknown side effects, addiction, and dependence on a medication.^38^ Additionally, other qualitative studies among Black persons suffering from depression revealed beliefs that, in the Black community, depression was a sign of weakness and persons should deal with his/her problems and not talk about their personal business with others.^39^, ^40^

Survivors of TTP expressed that surviving TTP was life altering and traumatic. The continued fatigue that patients are experiencing made it difficult for them to find motivation to do their daily activities, and as an extension, it may be difficult for patients to find the motivation to follow up on a referral to see about their depression. Patients also mentioned additional barriers for depression management, which included believing their depression was not severe enough for treatment and the financial costs of depression management. Of note, it was more commonly observed that Black patients described the belief that the residual effects of surviving TTP that they were dealing with (fear of relapsing, continued fatigue, and the unexpected mild cognitive impairment) was related to their depression. Our study was conducted in 2013; however, recently, a study reported that 32% of TTP survivors 3 to 11 years following their first TTP episode met criteria for a provisional diagnosis of post‐traumatic stress disorder related to their TTP. Additionally, of their participants who were unemployed, 53/82 (65%) of them subjectively attributed their unemployment to residual long‐term effects of TTP.^11^ This supports our finding that these patients are experiencing a traumatic life‐altering event that impacts them psychologically, well beyond the recovery of their platelet count. Finally, our patients expressed that the hematologist plays a vital role in their lives regarding depression management. This is not surprising, as a study with cancer survivors showed that oncologists' recommendations were influential to influencing a favorable attitude and positive social norms toward exercise.^41^

Limitations of our study include that qualitative studies are not designed to be generalizable to the larger population. Also, the majority of the TTP patients who were not interviewed were White, and if they had been included, the themes are key ideas could have changed. However, the qualitative nature of the study allowed us to gain an in‐depth understanding of patient perceptions of not only depression management but also the impact of TTP on their lives. Moreover, the intent of our study was to present the views of the TTP patients on depression management and not to validate the accuracy of the patients' beliefs and attitudes. We did not collect information on how long patients who were on pharmacotherapy had been taking the medication; therefore, patients were heterogeneous in their experiences. Our secondary research question on attitudes toward counseling was limited in that we did not systematically assess the history of counseling in our patients. As a result, some patients were likely reporting attitudes to counseling from personal experience, and others were reporting attitudes to counseling from vicarous experience of their friends and family. Another limitation is that we asked patients if they had a previous mental health diagnosis and patients could have said “no” to that question because of a social desirability bias or fear of stigma. Additionally, we did not explicitly ask about access to healthcare barriers. However, a few patients did mention that the cost of counseling sessions and/or pharmacologic treatment for depression was a potential barrier. Finally, our study was conducted in 2013, and views could have changed in the last few years. However, this study is currently the first study to look at attitudes to depression management among TTP patients.

The American College of Physicians recommends that clinicians manage major depressive disorder with either antidepressants or counseling after taking into account patient preferences.^42^ It has been shown that depression treatment acceptability is associated with treatment preference.^43^ Consequently, it is imperative that hematologists or primary care physicians begin to routinely screen for depression in these patients and make appropriate referrals when the patient suffers from moderate or major depression.^8^ The primary hematologist has a critical role, as they often serve the role of both a specialist and a primary care physician for these patients. Understanding management preferences will assist in tailoring discussions with the patient, and our results show that the majority of the TTP patients' attitudes towards pharmacologic management are similar to what has been found for persons with depression that have not recovered from TTP.

In conclusion, this is the first study to examine beliefs related to depression management in survivors of TTP. Our patients expressed that TTP was life altering and traumatic and that their physician plays a vital role in determining their depression management. Recognizing and ensuring effective depression management is a critical part of TTP follow‐up care. TTP care following recovery may need to follow a more integrated care approach to reach the needs of these patients.

## CONFLICTS OF INTEREST

The authors have no conflicts with this topic or the content of this manuscript.

## AUTHOR CONTRIBUTIONS

Conceptualization: Deirdra Terrell, Eleni Tolma, Lauren Stewart

Data curation: Deirdra Terrell, Lauren Stewart

Formal analysis: Deirdra Terrell, Eleni Tolma, Lauren Stewart, Erin Shirley

Methodology: Deirdra Terrell, Eleni Tolma

Project administration: Deirdra Terrell

Software: Erin Shirley

Supervision: Deirdra Terrell, Eleni Tolma

Validation: Deirdra Terrell

Writing – original draft preparation: Deirdra Terrell

Writing – review and editing: Deirdra Terrell, Eleni Tolma, Lauren Stewart, Erin Shirley

All authors have read and approved the final version of the manuscript. The corresponding author/manuscript guarantor (Deirdra R. Terrell) had full access to all of the data in this study and takes complete responsibility for the integrity of the data and the accuracy of the data analysis.

## TRANSPARENCY STATEMENT

Deirdra R. Terrell affirms that this manuscript is an honest, accurate, and transparent account of the study being reported; that no important aspects of the study have been omitted; and that any discrepancies from the study as planned (and, if relevant, registered) have been explained.

## FUNDING INFORMATION

This project was supported by the National Heart, Lung, and Blood Institute of the National Institutes of Health under award number 1K01HL135466. Dr Terrell is supported by a career development award from the National Heart, Lung, and Blood Institute (NHLBI). The NHLBI did not have any involvement in the study design, data collection, analysis, interpretation of the data, writing of the report, or the decision to submit the report for publication.

## DATA AVAILABILITY STATEMENT

The signed consent allows the use of interview transcripts for this study but not for further sharing. As such, transcripts are not currently available for secondary use.

## References

[1] Ridolfi RL , Bell WR . Thrombotic thrombocytopenic purpura. Report of 25 cases and review of the literature. Medicine. 1981;60(6):413‐428. 7031412

[2] Kremer Hovinga JA , Vesely SK , Terrell DR , Lammle B , George JN . Survival and relapse in patients with thrombotic thrombocytopenic purpura. Blood. 2010;115(8):1500‐1511. quiz 1662 20032506 10.1182/blood-2009-09-243790

[3] Coppo P , Veyradier A . Current management and therapeutical perspectives in thrombotic thrombocytopenic purpura. Presse Med. 2012;41(3 Pt 2):e163‐e176. 22265954 10.1016/j.lpm.2011.10.024

[4] Chiasakul T , Cuker A . Clinical and laboratory diagnosis of TTP: an integrated approach. Hematology Am Soc Hematol Educ Program. 2018;2018(1):530‐538. 30504354 10.1182/asheducation-2018.1.530 PMC6246034

[5] Blombery P , Scully M . Management of thrombotic thrombocytopenic purpura: current perspectives. J blood med. 2014;5:15‐23. 24523598 10.2147/JBM.S46458 PMC3921093

[6] Kremer Hovinga JA , Coppo P , Lammle B , Moake JL , Miyata T , Vanhoorelbeke K . Thrombotic thrombocytopenic purpura. Nat Rev Dis Primers. 2017;3(1):17020. 28382967 10.1038/nrdp.2017.20

[7] Reese JA , Muthurajah DS , Hovinga JAK , Vesely SK , Terrell DR , George JN . Children and adults with thrombotic thrombocytopenic purpura associated with severe, acquired Adamts13 deficiency: comparison of incidence, demographic and clinical features. Pediatr Blood Cancer. 2013;60(10):1676‐1682. 23729372 10.1002/pbc.24612

[8] Han B , Page EE , Stewart LM , et al. Depression and cognitive impairment following recovery from thrombotic thrombocytopenic purpura. Am J Hematol. 2015;90(8):709‐714. 25975932 10.1002/ajh.24060 PMC4509840

[9] Deford CC , Reese JA , Schwartz LH , et al. Multiple major morbidities and increased mortality during long‐term follow‐up after recovery from thrombotic thrombocytopenic purpura. Blood. 2013;122(12):2023‐2029. 23838348 10.1182/blood-2013-04-496752 PMC3778546

[10] Falter T , Schmitt V , Herold S , et al. Depression and cognitive deficits as long‐term consequences of thrombotic thrombocytopenic purpura. Transfusion. 2017;57(5):1152‐1162. 28337761 10.1111/trf.14060

[11] Chaturvedi S , Oluwole O , Cataland S , McCrae KR . Post‐traumatic stress disorder and depression in survivors of thrombotic thrombocytopenic purpura. Thromb Res. 2017;151:51‐56. 28113083 10.1016/j.thromres.2017.01.003

[12] Kessler RC , Bromet EJ . The epidemiology of depression across cultures. Annu Rev Public Health. 2013;34(1):119‐138. 23514317 10.1146/annurev-publhealth-031912-114409 PMC4100461

[13] Hardeveld F , Spijker J , De Graaf R , Nolen WA , Beekman AT . Prevalence and predictors of recurrence of major depressive disorder in the adult population. Acta Psychiatr Scand. 2010;122(3):184‐191. 20003092 10.1111/j.1600-0447.2009.01519.x

[14] Cuijpers P , Beekman AF , Reynolds CF . Preventing depression: a global priority. JAMA. 2012;307(10):1033‐1034. 22416097 10.1001/jama.2012.271 PMC3397158

[15] Cuijpers P , Smit F . Excess mortality in depression: a meta‐analysis of community studies. J Affect Disord. 2002;72(3):227‐236. 12450639 10.1016/s0165-0327(01)00413-x

[16] Smithson S , Pignone MP . Screening adults for depression in primary care. Med Clin North Am. 2017;101(4):807‐821. 28577628 10.1016/j.mcna.2017.03.010

[17] Kessler RC , Demler O , Frank RG , et al. Prevalence and treatment of mental disorders, 1990 to 2003. New Engl J Med. 2005;352(24):2515‐2523. 15958807 10.1056/NEJMsa043266 PMC2847367

[18] Norquist GS , Regier DA . The epidemiology of psychiatric disorders and the de facto mental health care system. Annu Rev Med. 1996;47(1):473‐479. 8712797 10.1146/annurev.med.47.1.473

[19] Berkowitz SA , Bell RA , Kravitz RL , Feldman MD . Vicarious experience affects patients' treatment preferences for depression. Plos One. 2012;7(2):e31269. 22363603 10.1371/journal.pone.0031269 PMC3283627

[20] Fishbein M , Ajzen I . . Belief, Attitude, Intention and Behavior: An Introduction to Theory and Research Reading: Addison‐Wesley; 1975.

[21] Zivin K , Kales HC . Adherence to depression treatment in older adults: a narrative review. Drugs Aging. 2008;25(7):559‐571. 18582145 10.2165/00002512-200825070-00003

[22] Van Voorhees BW , Fogel J , Houston TK , Cooper LA , Wang NY , Ford DE . Beliefs and attitudes associated with the intention to not accept the diagnosis of depression among young adults. Ann Fam Med. 2005;3(1):38‐46. 15671189 10.1370/afm.273 PMC1466793

[23] Cooper LA , Gonzales JJ , Gallo JJ , et al. The acceptability of treatment for depression among African‐American, Hispanic, and White primary care patients. Med Care. 2003;41(4):479‐489. 12665712 10.1097/01.MLR.0000053228.58042.E4

[24] Guest G , Bunce A , Johnson L . How many interviews are enough?: an experiment with data saturation and variability. Field Methods. 2006;18(1):59‐82.

[25] Montano DE KD . The Theory of Reasoned Action and the Theory of Planned Behavior In: Glanz K RB , Lewis FM, ed. Health Behavior and Health Education: Theory, Research and Practice Vol 3rd. San Francisco: Jossey‐Bass; 2002:67‐99.

[26] Saunders B , Sim J , Kingstone T , et al. Saturation in qualitative research: exploring its conceptualization and operationalization. Qual Quant. 2018;52(4):1893‐1907. 29937585 10.1007/s11135-017-0574-8 PMC5993836

[27] Rock GA , Shumak KH , Buskard NA , et al. Comparison of plasma exchange with plasma infusion in the treatment of thrombotic thrombocytopenic purpura. Canadian Apheresis Study Group. N Engl J Med. 1991;325(6):393‐397. 2062330 10.1056/NEJM199108083250604

[28] Vesely SK , George JN , Lammle B , et al. ADAMTS13 activity in thrombotic thrombocytopenic purpura‐hemolytic uremic syndrome: relation to presenting features and clinical outcomes in a prospective cohort of 142 patients. Blood. 2003;102(1):60‐68. 12637323 10.1182/blood-2003-01-0193

[29] Page EE , Kremer Hovinga JA , Terrell DR , Vesely SK , George JN . Thrombotic thrombocytopenic purpura: diagnostic criteria, clinical features, and long‐term outcomes from 1995 through 2015. Blood advances. 2017;1(10):590‐600. 29296701 10.1182/bloodadvances.2017005124 PMC5728353

[30] Beck AT , Steer RA , Ball R , Ranieri W . Comparison of Beck Depression Inventories ‐IA and ‐II in psychiatric outpatients. J Pers Assess. 1996;67(3):588‐597. 8991972 10.1207/s15327752jpa6703_13

[31] Kroenke K , Spitzer RL , Williams JBW . The PHQ‐9: Validity of a brief depression severity measure. J Gen Intern Med. 2001;16(9):606‐613. 11556941 10.1046/j.1525-1497.2001.016009606.x PMC1495268

[32] Patton MQ . Qualitative Research & Evaluation Methods. 3rd ed. Thousand Oaks, California: Sage Publications; 2002:230‐234.

[33] Sclar DA , Robison LM , Schmidt JM , Bowen KA , Castillo LV , Oganov AM . Diagnosis of depression and use of antidepressant pharmacotherapy among adults in the United States: does a disparity persist by ethnicity/race? Clin Drug Investig. 2012;32(2):139‐144. 10.2165/11598950-000000000-00000 22220929

[34] Breslau J , Cefalu M , Wong EC , et al. Racial/ethnic differences in perception of need for mental health treatment in a US national sample. Soc Psychiatry Psychiatr Epidemiol. 2017;52(8):929‐937. 28550518 10.1007/s00127-017-1400-2 PMC5534379

[35] Ulin PRRE , Tolley EE . Qualitative Methods in Public Health: A Field Guide for Applied Research. 1st ed. San Francisco, CA: Jossey‐Bass; 2005.

[36] Tong A , Sainsbury P , Craig J . Consolidated criteria for reporting qualitative research (COREQ): a 32‐item checklist for interviews and focus groups. Int J Qual Health Care: J Int Soc Qual Health Care. 2007;19(6):349‐357. 10.1093/intqhc/mzm042 17872937

[37] Goodwin GM , Price J , De Bodinat C , Laredo J . Emotional blunting with antidepressant treatments: a survey among depressed patients. J Affect Disord. 2017;221:31‐35. 28628765 10.1016/j.jad.2017.05.048

[38] Waite R , Killian P . Perspectives about depression: explanatory models among African‐American women. Arch Psychiatr Nurs. 2009;23(4):323‐333. 19631110 10.1016/j.apnu.2008.05.009

[39] Conner KO , Copeland VC , Grote NK , et al. Barriers to treatment and culturally endorsed coping strategies among depressed African‐American older adults. Aging Ment Health. 2010;14(8):971‐983. 21069603 10.1080/13607863.2010.501061 PMC3060025

[40] Bryant K , Greer‐Williams N , Willis N , Hartwig M . Barriers to diagnosis and treatment of depression: voices from a rural African‐American faith community. J National Black Nurses' Assoc JNBNA. 2013;24(1):31‐38. PMC3983966 24218871

[41] Jones LW , Courneya KS , Fairey AS , Mackey JR . Does the theory of planned behavior mediate the effects of an oncologist's recommendation to exercise in newly diagnosed breast cancer survivors? Results from a randomized controlled trial. Health Psychol. 2005;24(2):189‐197. 15755233 10.1037/0278-6133.24.2.189

[42] Qaseem A , Barry MJ , Kansagara D . Nonpharmacologic versus pharmacologic treatment of adult patients with major depressive disorder: a clinical practice guideline from the American College of Physicians. Ann Intern Med. 2016;164(5):350‐359. 26857948 10.7326/M15-2570

[43] Houle J , Villaggi B , Beaulieu MD , Lesperance F , Rondeau G , Lambert J . Treatment preferences in patients with first episode depression. J Affect Disord. 2013;147(1‐3):94‐100. 23167975 10.1016/j.jad.2012.10.016

